# The plasticity of neuropeptide Y-Y1 receptor system on *Tac2* neurons contributes to mechanical hyperknesis during chronic itch

**DOI:** 10.7150/thno.89433

**Published:** 2024-01-01

**Authors:** Danqing Dai, Tiantian Zhao, Zhen Li, Wanrong Li, Aiwen Chen, Yali Tang, Xiao-Fei Gao, Lize Xiong

**Affiliations:** 1 Shanghai Key Laboratory of Anesthesiology and Brain Functional Modulation, Clinical Research Center for Anesthesiology and Perioperative Medicine, Translational Research Institute of Brain and Brain-Like Intelligence, Shanghai Fourth People's Hospital, School of Medicine, Tongji University, No.1481, Xinshi North Road, Shanghai 200434, China.; 2 Department of Anesthesiology and Perioperative Medicine, Shanghai Fourth People's Hospital, School of Medicine, Tongji University, No. 1279, Sanmen Road, Shanghai 200434, China.

**Keywords:** itch, spinal cord, neuropeptide, NPY, Y1 receptor

## Abstract

**Rationale:** In the physiological states, the act of scratching protects the person from harmful substances, while in certain pathological conditions, the patient suffers from chronic itch, both physically and mentally. Chronic itch sufferers are more sensitive to mechanical stimuli, and mechanical hyperknesis relief is essential for chronic itch treatment. While neuropeptide Y-Y1 receptor (NPY-Y1R) system is known to play a crucial role in modulating mechanical itch in physiological conditions, it is elusive how they are altered during chronic itch. We hypothesize that the negative regulatory effect of Y1Rs on *Tac2* neurons, the key neurons that transmit mechanical itch, declines during chronic itch.

**Methods:** We combined transgenic mice, chemogenetic manipulation, immunofluorescence, rabies virus circuit tracing, and electrophysiology to investigate the plasticity of Y1Rs on *Tac2* neurons during chronic itch.

**Results:** We found that *Tac2* neurons receive direct input from *Npy* neurons and that inhibition of *Npy* neurons induces activation of *Tac2* neurons. Moreover, the expression of Y1Rs on *Tac2* neurons is reduced, and the regulatory effect is also reduced during chronic itch.

**Conclusion:** Our study clarifies the plasticity of Y1Rs on *Tac2* neurons during chronic itch and further elucidates the mechanism by which NPY-Y1R system is responsible for modulating mechanical itch. We highlight Y1Rs as a promising therapeutic target for mechanical hyperknesis during chronic itch.

## Introduction

Itching is an unpleasant somatosensory sensation that elicits scratching behavior. Mechanical or chemical itch signals are transmitted from the skin to distinct primary sensory neurons 1. Under normal physiological conditions, scratching aids in removing potentially harmful substances from the skin and serves as a protective mechanism for animals. However, in certain pathological conditions such as atopic dermatitis and psoriasis, patients suffer from excessive scratches that not only severely damage the skin but also lead to sleep disorders, anxiety, and depression, significantly compromising their quality of life 2-5. This abnormal itch often persists for more than 6 weeks and is referred to as chronic itch 6. Patients with chronic itch demonstrate heightened sensitivity to external chemical and mechanical stimuli. Exogenous chemical pruritogens commonly encompass insect bites, plant sap and cosmetics. Conversely, patients with chronic itch experience a greater prevalence of mechanical stimuli such as light touch from clothing or movement of insects on the skin. Mechanical itch is distinguished by hyperknesis and alloknesis, denoting an augmented perception of itch in response to typically pruritic stimuli and itch provoked by gentle cutaneous stimuli like clothing respectively 7, 8. Alleviating mechanical itch assumes paramount importance in the treatment of chronic itch.

So far, two spinal pathways responsible for transmitting mechanical itch have been identified. Firstly, Bo Duan *et al.* reported a pathway involving “TLR5^+^ neurons →* Ucn3* neurons → ascending projection neurons" for the transmission of mechanical itch 9. They discovered that a group of excitatory interneurons expressing urocortin 3 (Ucn3) in the spinal cord are involved in acute mechanical itch transmission. Ablation or silencing of these neurons resulted in reduced mechanical itch sensation, while specific activation of these neurons induced spontaneous itch. Their study also revealed that toll-like receptor 5-positive (TLR5^+^) Aβ fibers originating from the dorsal root ganglion project to *Ucn3* neurons in the spinal cord. Secondly, another pathway termed "LTMR → *Tac2* neurons → *Grpr* neurons → ascending projection neurons" was identified 10. *Tac2* neurons are primarily located in lamina IIi and IIIo of the spinal cord, where they receive direct input from Aβ low threshold mechanoreceptors (LTMRs) 10, 11. Activation of *Tac2* neurons in the spinal cord was observed through an increase in c-Fos expression following mechanical itch stimulation. Optogenetic activation of* Tac2* neurons elicited scratching behavior, while ablation or silencing of these neurons attenuated mechanical itch. These findings lend support to the notion that *Tac2* neurons possess the ability to detect innocuous mechanical stimuli and transmit signals associated with mechanical itch 10.

On the other hand, considerable efforts have been devoted to modulating mechanical itch. Among the most promising targets for modulation lies neuropeptide Y (NPY) and its receptor NPY receptor 1 (NPY1R). It has been demonstrated that spinal cord neurons expressing NPY and Y1Rs exert control over pain sensory input 12-18. Recent investigations have also highlighted the involvement of NPY and Y1Rs in regulating itch. For instance, intrathecal administration of NPY has exhibited inhibitory effects on morphine-induced spontaneous itch 19, while intrathecal administration of the Y1R agonist [Leu^31^, Pro^34^]-NPY (LP-NPY) has shown attenuation of both mechanical itch induced by a 0.07 g von Frey filament and chemical itch induced by histamine 20. Chemogenetic activation of *Npy* neurons leads to a reduction in pruritogen-evoked chemical itch, while toxin-mediated silencing of *Npy* neurons induces spontaneous itch and enhances pruritogen-evoked itch 21. Moreover, the observed mechanical hyperknesis in aged mice can be attributed to a deficiency of spinal NPY 22, as well as the ablation or silencing of spinal *Npy* neurons or conditional knockout of spinal Y1Rs in mice, which induce mechanical hyperknesis 1, 23. Additionally, Bo Duan *et al.* successfully incorporated NPY-Y1R into the neuronal circuits responsible for mechanical itch and demonstrated that *Npy* neurons regulate mechanical itch by inhibiting *Ucn3* neurons 9. Although it is known that NPY-Y1R plays a crucial role in modulating physiological conditions associated with mechanical itch, our understanding regarding their alterations during chronic itch remains limited.

Given that *Tac2* neurons are a specific subset of *Ucn3* neurons located in lamina II-III and potentially represent a more precise neural circuit for mechanical itch 10, we selected *Tac2* neurons as the target cells for our investigation. The objective of this study was to elucidate the plasticity of Y1Rs on *Tac2* neurons during chronic itch, aiming to provide a comprehensive understanding of their role in this pathological condition.

## Materials and Methods

### Animals

All procedures were approved by the Animal Research Ethics Committee of Tongji University (approval number TJBH00721101). The animals used in the present study include adult male (8-12 weeks old) C57BL/6J mice (SPF Biotech, Beijing), Ai9 (Jackson Laboratory, Stock No. 007909), *Npy^flpo^* (Jackson Laboratory, Stock No. 030211) and *Tac2^cre^* transgenic mice (Jackson Laboratory, Stock No. 018938). All mice were housed under a 12 h light/dark cycle (light on from 7 AM to 7 PM) at 22-25 °C with food and water ad libitum.

### Viruses

The following viruses were purchased from Brain VTA Co., Ltd. (Wuhan, China): AAV2/9-hSyn-DIO-EGFP-WPRE-hGH pA (titer: 5.04 × 10^12^ genomic copies per ml), AAV2/9-hSyn-fDIO-mCherry-WPRE-hGH pA (titer: 5.60 × 10^12^ genomic copies per ml), AAV2/9-hSyn-fDIO-hM4Di-mCherry-WPRE-hGH pA (titer: 5.19 × 10^12^ genomic copies per ml). For retrograde monosynaptic tracing, AAV2/9-EF1a-DIO-mCherry-F2A-TVA-WPRE-hGH pA (titer: 5.33 × 10^12^ genomic copies per ml), AAV2/9-EF1a-DIO-oRVG-WPRE-hGH pA (titer: 5.27 × 10^12^ genomic copies per ml), and RV-ENVA-ΔG-EGFP (titer: 2 × 10^8^ genomic copies per ml) were also packaged by Brain VTA Co., Ltd. (Wuhan, China). All viral vectors were aliquoted and stored at -80 °C until use.

### Intraspinal virus injection and chemogenetics

Stereotaxic surgery was performed as previously described 24. Mice were anesthetized with 1-1.5% isoflurane and arranged in a stereotaxic instrument (RWD Apparatus, Shenzhen, China). Animal eyes were coated with erythromycin eye ointment. Body temperature was kept stable with an electric heating pad. The cervical vertebrae were exposed at C2-C6, and the vertebral column was mounted onto a stereotaxic frame with spinal adapters. The virus was injected unilaterally with a glass pipette (10-20 μm in diameter at the tip) connected to a 10 μl microliter syringe (Gaoge, Shanghai, China). For example, AAV2/9-hSyn-DIO-EGFP with AAV2/9-hSyn-fDIO-mCherry or AAV2/9-hSyn-fDIO-hM4Di-mCherry was injected into the right side of the spinal cord at 3-4 sites between successive vertebrae at C2-C6. The glass pipette tip was inserted into the dorsal spinal cord vertically at a depth of 300-400 μm to target the lamina II-III. The AAV was injected (~400 nl per injection) at a rate of 40 nl/min with a syringe pump (KD Scientific, Cat# 788130). The micropipette was withdrawn 5 min later. After surgery, the animals were allowed to recover from anesthesia on an electric heating pad. Three or four weeks were allowed for virus expression before CNO injection. Successful injection of viruses was verified by post hoc immunofluorescence. Behavioral experiments were performed 30-40 min after the CNO application (5 mg/kg, intraperitoneal injection). All those animals with incorrect injection positions were excluded.

### Rotarod test

Mice were placed on a rotarod apparatus that accelerates 5-20 revolution per min (r.p.m.) for 5 min and trained to maintain their balancing walking on the first two days. On the third day, rod accelerated 5-40 r.p.m. and mice were tested three times with 10 min intervals (cut-off time 300 s). The latencies of mice to fall off were recorded for analysis.

### Open field test

Locomotor activity of mice was evaluated by open field test over a 10 min period in (50 × 50 × 50 cm) an apparatus. Mice were placed in center of the box and were videotaped individually. The area was cleaned with 75% ethanol after each test to remove olfactory cues from the apparatus.

### Mechanical itch test

We shaved the nape of mice 3 days before the experiment. Animals were placed in a plastic chamber and acclimatized for at least 2 days before the behavioral test. For DCP group mice, mechanical stimuli on the nape were delivered with von Frey filaments ranging from 0.008 to 1.0 g. For the *Npy^flpo^* neurons silencing experiment, mice were acclimatized for 30 min and then briefly removed from the chamber for intraperitoneal injection of CNO (5 mg/kg) on the day of the experiment. All animals received an injection of CNO. Mice were then returned to the chamber. 30 to 40 min after CNO injection, which coincides with the time of maximal neuronal silencing, each mouse received 5 separate mechanical stimuli at 10 s intervals delivered with von Frey filaments (0.04 g and 0.07 g) at the right side of the nape. Experiments with high intensity von Frey filaments (0.6 g and 1.0 g) were performed 2 days later as previously described. Positive responses were counted as hindlimb scratching towards the site of mechanical stimulation. The number of scratch episodes was the total number of positive responses elicited by 5 stimuli 1.

### Immunofluorescence

Mice were deeply anesthetized with isoflurane and perfused intracardially with 4% paraformaldehyde in PBS. Tissues were dissected, post-fixed overnight, and cryoprotected in 30% sucrose-PBS overnight at 4 °C. The cervical spinal cords were embedded in optimal cutting temperature compound (OCT, 4583, Tissue-Tek, SAKURA, Torrance, CA). 30 μm-thick sections were cut with a cryomicrotome (CM 1950, Leica Microsystems, Nussloch, Germany). Free-floating frozen sections were blocked for 2 h at room temperature with PBS containing 5% goat serum followed by incubation with primary antibodies in 0.1% PBST overnight at 4 °C. Primary antibodies utilized in this study included: rabbit polyclonal antibody to Neuropeptide Y1 receptor (1:300 dilution, RA24506-100, Neuromics) and guinea pig polyclonal antibody to c-Fos (1:1000 dilution, 226 004, Synaptic systems). And secondary antibodies for 2 h at 4 °C. The following secondary antibodies were used: goat polyclonal secondary antibody to rabbit IgG-H&L (Alexa Fluor 488, 1:500 dilution, ab150077, Abcam, Cambridge, UK) and goat polyclonal secondary antibody to guinea pig IgG-H&L (Alexa Fluor 647, 1:500 dilution, ab150187, Abcam, Cambridge, UK). Sections were stained with DAPI for 1 min (1 mg/ml dissolved in double steaming water for stock, and 10 μg/ml diluted with PBS as working concentration, 1155, BioFroxx, Einhausen, Germany) and sealed with an appropriate amount of antifading mounting medium (S2100, Solarbio, Shanghai, China). Fluorescent Images were captured with an Olympus FluoView FV3000 self-contained confocal laser scanning microscope system (Olympus, Tokyo, Japan). The representative images of immunofluorescence were taken from at least 3 mice.

### PinpoRNA^TM^ Multiplex Fluorescent RNA *in-situ* hybridization kit

The spinal cord sections were handled according to the manufacturer's instructions in the PinpoRNA^TM^ Multiplex Fluorescent Assay manual for fixed frozen tissue, and coverslipped with antifading mounting medium (S2100, Solarbio, Shanghai, China). A series of short probes (Cat# 1096481-B1) sequentially complementary to *Npy* target RNA sequence covering region 10-567 was designed by PinpoRNA (nucleotide target region: 29 → 64; GenBank: NM_023456.3). Briefly, the sections were fixed by 4% PFA and then the endogenous peroxidase was inhibited by Pre-A solution at room temperature. Target RNA molecules were exposed by protease treatment and hybridized with probes for 2 h at 40 °C. Then the signal was amplified sequentially by reaction 1, 2 and 3 while HRP molecule was lastly added into reaction 3. For the last step, a tyramide fluorescent substrate (OpalTM690, Akoya Biosciences) was added and target RNA was labeled by Tyramide Signal Amplification (TSA) assay. Sections were subsequently imaged under an Olympus FluoView FV3000 self-contained confocal laser scanning microscope (Olympus, Tokyo, Japan) in four channels with a 20X objective lens.

### Monosynaptic retrograde tracing

Briefly, 400 nl of the mixture of AAV2/9-EF1a-DIO-mCherry-F2A-TVA-WPRE-hGH pA (titer: 5.33 × 10^12^ genomic copies per ml) and AAV2/9-EF1a-DIO-oRVG-WPRE-hGH pA (titer: 5.27 × 10^12^ genomic copies per ml, BrainVTA Co., Ltd., Wuhan, China) (volume ratio: 1:1) was injected into the cervical spinal cord of *Tac2^Cre^* mice. Four weeks later, the spinal cord of mice was injected with 200 nl of RV-ENVA-ΔG-EGFP (titer: 2 × 10^8^ genomic copies per ml, BrainVTA Co., Ltd., Wuhan, China) at the same site. One week later, the mice were perfused, and the spinal cord was sectioned and imaged under an Olympus FluoView FV3000 self-contained confocal laser scanning microscope (Olympus, Tokyo, Japan). The sections were then used for PinpoRNA^TM^ Multiplex Fluorescent RNA *in-situ* hybridization using *Npy* probe and imaged under the confocal microscope. Images taken before and after *in-situ* hybridization were aligned and merged for analysis by Photoshop software (Adobe Photoshop CC 2018).

### Electrophysiology

Adult mice (8-12 weeks old) were deeply anesthetized with isoflurane. Then they were perfused with 30 ml ice-cold (4 °C) NMDG slicing solution (in mM, 93 NMDG, 2.5 KCl, 1.25 NaH_2_PO_4_, 30 NaHCO_3_, 20 HEPES, 25 Dextrose, 12.1 N HCl, 5 Ascorbic acid, 2 Thiourea, 3 Na^+^ pyruvate, 10 MgSO_4_, 0.5 CaCl_2_, 12 N-acetylcysteine, pH was adjusted to 7.3-7.4 with NMDG). Spinal cord was isolated under oxygenated (95% O_2_, 5% CO_2_) sucrose-based dissection solution (in mM, 209 Sucrose, 2 KCl, 1.25 NaH_2_PO_4_, 5 MgCl_2_, 0.5 CaCl_2_, 26 NaHCO_3_, 10 Dextrose, pH was adjusted to 7.3-7.4) and the cervical region was embedded in agar. Sections of the cervical cord were obtained at 300 µm using a vibrating slicer (LEICA VT1000S). The slices were recovered in a chamber containing 37 °C oxygenated holding solution (in mM, 92 NaCl, 2.5 KCl, 1.25 NaH_2_PO_4_, 30 NaHCO_3_, 20 HEPES, 25 Dextrose, 2 MgCl_2_, 2 CaCl_2_, pH was adjusted to 7.3-7.4) for 1 h. Neurons were visualized with 593 nm light (TXRED filter) under an upright microscope (Olympus BX 51WI). Slices were placed in a chamber and perfused with oxygenated ACSF at 2 ml/min (in mM, 124 NaCl, 2.5 KCl, 1.25 NaH_2_PO_4_, 24 NaHCO_3_, 5 HEPES, 12.5 Dextrose, 1 MgCl_2_, 2 CaCl_2_, pH was adjusted to 7.3-7.4). Patch pipettes (BF150-86-10, Sutter Instrument, Novato, CA) were pulled to a resistance of 4-8 MΩ by a horizontal puller (P-97, Sutter Instrument, Novato, CA). Signals were amplified with Multiclamp 700B and Digidata 1550A and pClamp 10.7 software (Molecular Devices). Signals were filtered at 2 kHz and digitized at 20 kHz.

*Tac2* neurons were recorded by an electrode filled with normal pipette solution (in mM, 130 K gluconate, 10 NaCl, 0.2 EGTA, 10 HEPES, 1 MgATP, 5 Na_2_GTP, 1 MgCl_2_, pH was adjusted to 7.2). After whole-cell configuration, the resting membrane potential, membrane capacitance and series resistance were recorded immediately. If the resting membrane potential was positive to -50 mV, the data were excluded.

APs were recorded in current-clamp mode at the resting membrane potential and in response to a series of current injections with 10 pA increments. To avoid potential damage to neurons, we applied step stimuli of less than 80 pA to the neurons. If 80 pA could not elicit action potential, we record the rheobase as 100 pA. To examine the change in membrane conductance of *Tac2* neurons in response to LP-NPY, we recorded the LP-NPY-induced outward currents at the holding potential of -45 mV. The miniature excitatory postsynaptic currents (mEPSCs) and miniature inhibitory postsynaptic currents (mIPSCs) were recorded at the holding potential of -70 or 0 mV, respectively in the presence of tetrodotoxin (TTX, 1 μM). Data were analyzed with Clampfit 10.6 (Axon Instruments, Foster City, CA), Mini Analysis Program 6.0.3 (Synaptosoft) and GraphPad Prism 8 software (GraphPad, San Diego, CA). Traces were plotted using Origin software (OriginLab, Northampton, MA).

### Administration of Drugs

Drugs dissolved in ACSF were applied via a three-way stopcock without changing the perfusion rate and temperature. A change in the solution of the recording chamber completes within 20 s. The drugs used in this study were [Leu^31^, Pro^34^]-NPY (1 μM, Tocris) and TTX (1 μM, Taoto Biotech). Drug solutions were given by a drug delivery system (MVC-801, MappingLab, UK).

### Diphenylcyclopropenone (DCP)-induced chronic itch model

A contact dermatitis model of chronic itch was made by applying DCP onto the neck skin as previously described 25. Briefly, mice were shaved on the neck and the shaved area was topically applied 0.2 ml DCP (1% dissolved in acetone) for initial sensitization. Seven days after the first painting, the treated area was challenged by painting with 0.2 ml 0.5% DCP for 7 days. Electrophysiological experiments are performed after the final DCP application. The timelines for DCP treatment and behavioral measurements are illustrated in Figure 3A.

### Statistical Analysis

The statistical analyses were performed using Graphpad Prism 8 software (GraphPad, San Diego, CA). All data are presented as mean ± standard error of the mean (s.e.m.) and checked normality before analysis. For parametric comparison between two groups, an F-test was conducted to determine the similarity in the variances between the groups, and statistical significance was analyzed using the Student's *t*-test. For multiple comparisons, two-way analysis of variance (ANOVA) followed by Sidak's multiple comparisons test was used to test statistical significance. For statistical analysis of the ratio of* Tac2* and Y1R double staining neurons, as well as the ratio of *Tac2* and c-Fos double staining neurons, data were analyzed with Chi-square test. For statistical analysis of the incidence of electrophysiological results, data were analyzed with Fisher's exact test. For statistical analysis of cumulative fraction, K-S test was used. A *p* value less than 0.05 was accepted as statistically significant.

## Results

### Inhibition of spinal *Npy* neurons induces hyperknesis and activation of *Tac2* neurons

**Figure 1:**
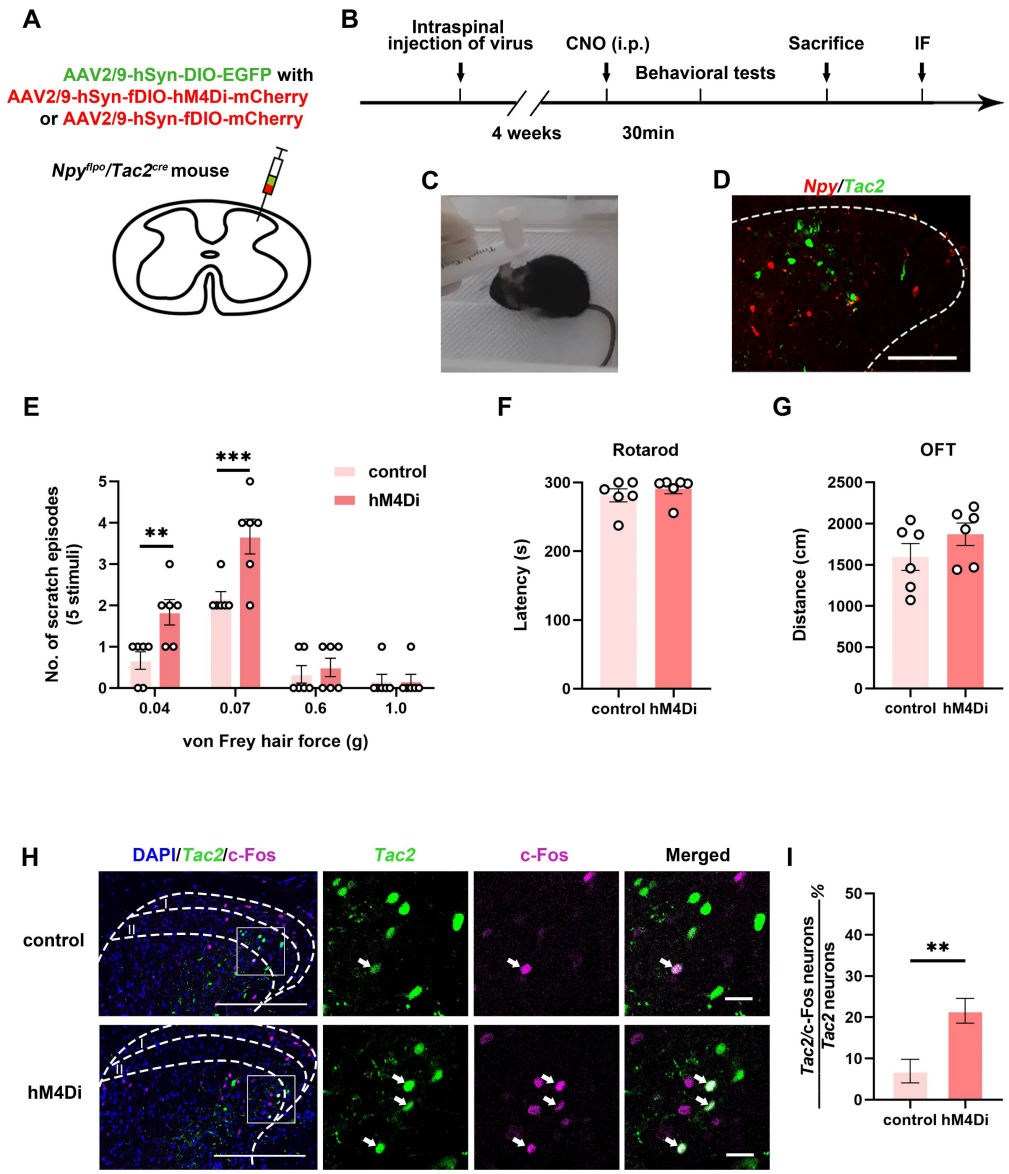
** Inhibition of spinal *Npy* neurons induces hyperknesis and activation of *Tac2* neurons.** (A) A diagram showing unilateral injection of AAV2/9-hSyn-DIO-EGFP and AAV2/9-hSyn-fDIO-hM4Di-mCherry (hM4Di group) or AAV2/9-hSyn-fDIO-mCherry (control group) virus into the cervical dorsal spinal cord of *Npy^flpo^/Tac2^cre^* mice. (B) A timeline of behavioral experiments. (C) A snapshot of mouse scratching induced by von Frey filament. (D) Immunofluorescence image of *Npy* neurons (red) and *Tac2* neurons (green) in the cervical spinal cord. Scale bar, 80 μm. (E) An increase in scratches to low intensity (0.04 and 0.07 g) but not high intensity force (0.6 and 1.0 g) is seen in hM4Di group compared with control group. Two-way ANOVA, Sidak's multiple comparisons test, *p*_0.04_ = 0.0077, *p*_0.07_ = 0.0005, *p*_0.6_ = 0.9828, *p*_1.0_ > 0.9999. (F) and (G) Chemogenetic inhibition of *Npy* neurons did not significantly affect motor coordination as tested by rotarod (F), or locomotor activity as tested by open field test (G). n_control_ = 6 mice, n_hM4Di_ = 6 mice, two-tailed unpaired Student's *t*-test, *p*_rotarod_ = 0.4455, *p*_OFT_ = 0.2207. (H) Expression of c-Fos in *Tac2* neurons in the control and hM4Di groups by immunostaining. Arrow indicates double-stained *Tac2*/c-Fos neurons. Scale bars, 200 μm in DAPI/*Tac2*/c-Fos column, 20 μm in *Tac2*, c-Fos and Merged columns. (I) Percentages of double-stained *Tac2*/c-Fos neurons in* Tac2* neurons in control (6.9 ± 2.9%) and hM4Di (21.6 ± 3%) groups. n_control_ = 76 neurons, n_hM4Di_ = 67 neurons; *p* = 0.0035, Chi-square test.

To figure out the inhibitory modulatory role of spinal *Npy* neurons in the mechanical itch sensation, we injected AAV2/9-hSyn-DIO-EGFP with AAV2/9-hSyn-fDIO-hM4Di-mCherry virus (hM4Di group) or AAV2/9-hSyn-fDIO-mCherry virus (control group) into the cervical spinal cord of *Npy^flpo^*/*Tac2^cre^* mice (Figure 1A-B). Thirty minutes after intraperitoneal injection of clozapine-N-oxide (CNO) (5 mg/kg), we measured the numbers of scratch episodes in response to 5 stimuli of each von Frey filament (0.04, 0.07, 0.6 and 1.0 g) (Figure 1C). The successful injections of viruses were verified by post-hoc immunofluorescence (Figure 1D) and patch clamp recording (Figure S1). We found that innocuous mechanical stimuli (0.04 and 0.07 g) induced more scratches in the hM4Di group than the control group (0.04 g: control 0.7 ± 0.2 scratches, hM4Di 1.8 ± 0.3 scratches, *p* = 0.0077; 0.07 g: control 2.2 ± 0.2 scratches, hM4Di 3.7 ± 0.4 scratches,* p* = 0.0005). While there is no difference in the numbers of scratch episodes between control group and hM4Di group in response to noxious mechanical stimuli (0.6 and 1.0 g) that typically produce pain (0.6 g: control 0.3 ± 0.2 scratches, hM4Di 0.5 ± 0.2 scratches, *p* = 0.9828; 1.0 g: control 0.2 ± 0.2 scratches, hM4Di 0.2 ± 0.2 scratches, *p* > 0.9999) (Figure 1E). Otherwise, silence of the* Npy* neurons did not affect the motor coordination or locomotor activity (Figure 1F-G). Our results indicate inhibition of spinal *Npy* neurons induces hyperknesis which is in line with an earlier report 1. To further determine the activity of *Tac2* neurons when inhibiting *Npy* neurons, we used c-Fos, a neuronal activity marker. Compared with the control group (6.6%, 5/76), silence of *Npy* neurons elicited more c-Fos expression in *Tac2* neurons (23.9%, 16/67, *p* = 0.0043), revealing disinhibition of *Tac2* neurons (Figure 1H-I). Therefore, inhibition of spinal *Npy* neurons induced activation of *Tac2* neurons.

### *Tac2* neurons receive direct inputs from *Npy* neurons

**Figure 2:**
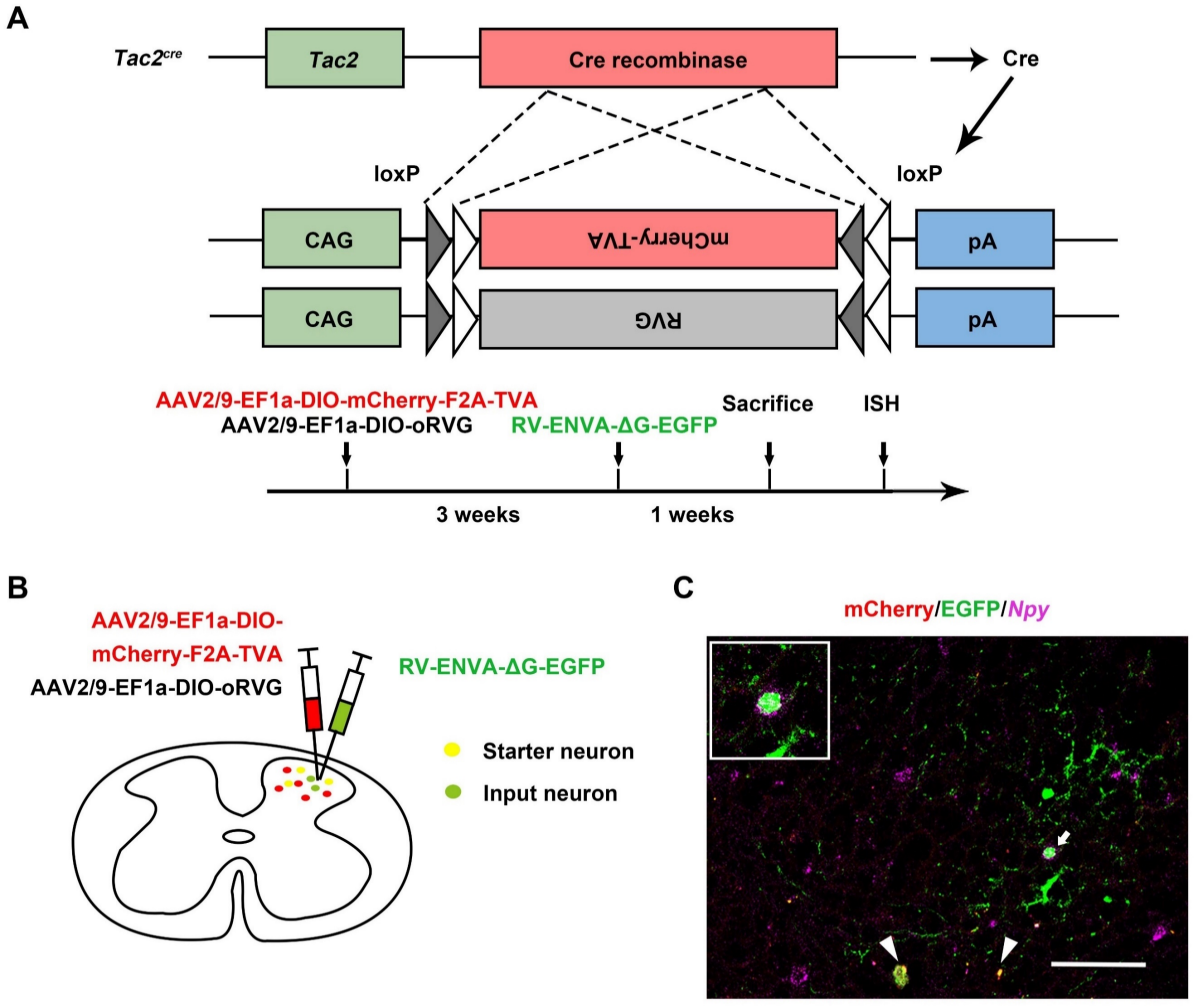
***Tac2* neurons receive direct inputs from *Npy* neurons.** (A) Mechanism and timeline of the rabies virus tracing experiment. (B) A schematic diagram of intraspinal injection of AAV2/9-EF1a-DIO-mCherry-F2A-TVA, AAV2/9-EF1a-DIO-oRVG viruses and RV-ENVA-ΔG-EGFP virus. (C) Representative ISH image of *Npy* (purple) in the cervical spinal cord after RVΔG injections. Arrowheads indicate *Tac2* starter neurons (yellow) expressing mCherry (red) and EGFP (green). Arrow indicates starter neurons targeting* Npy* neurons (EGFP^+^ and purple^+^) which is enlarged on the upper left corner. Scale bar, 80 μm.

Given that spinal *Tac2* neurons transmit mechanical itch 10 and silence of *Npy* neurons elicited disinhibition of *Tac2* neurons, we wondered whether *Npy* neurons connect with *Tac2* neurons directly. We employed rabies virus circuit tracing method, using *Tac2^cre^* mice with glycoprotein-deleted rabies virus (RVΔG virus). The cervical spinal cord was injected with mixed helper AAV viruses namely AAV2/9-EF1a-DIO-mCherry-F2A-TVA and AAV2/9-EF1a-DIO-oRVG virus to label *Tac2* neurons with mCherry and the glycoprotein of RV (RVG). Three weeks later, the RV-ENVA-ΔG-EGFP virus was injected into the same area to infect the mCherry-labeled TVA-expressing *Tac2* neurons (Figure 2A). Neurons coexpressing mCherry and EGFP are identified as starter cells. Assisted with RVG in the starter neurons, RVΔG would retrogradely label the input neurons with EGFP (Figure 2B). Examination of *Npy* mRNA (purple) with PinpoRNA^TM^ Multiplex Fluorescent RNA *in-situ* hybridization showed that the input neurons that target *Tac2* neurons express *Npy* (Figure 2C), indicating the existence of monosynaptic connections between *Npy* neurons and *Tac2* neurons.

### Y1 receptor expression in *Tac2* neurons decreases during chronic itch

**Figure 3:**
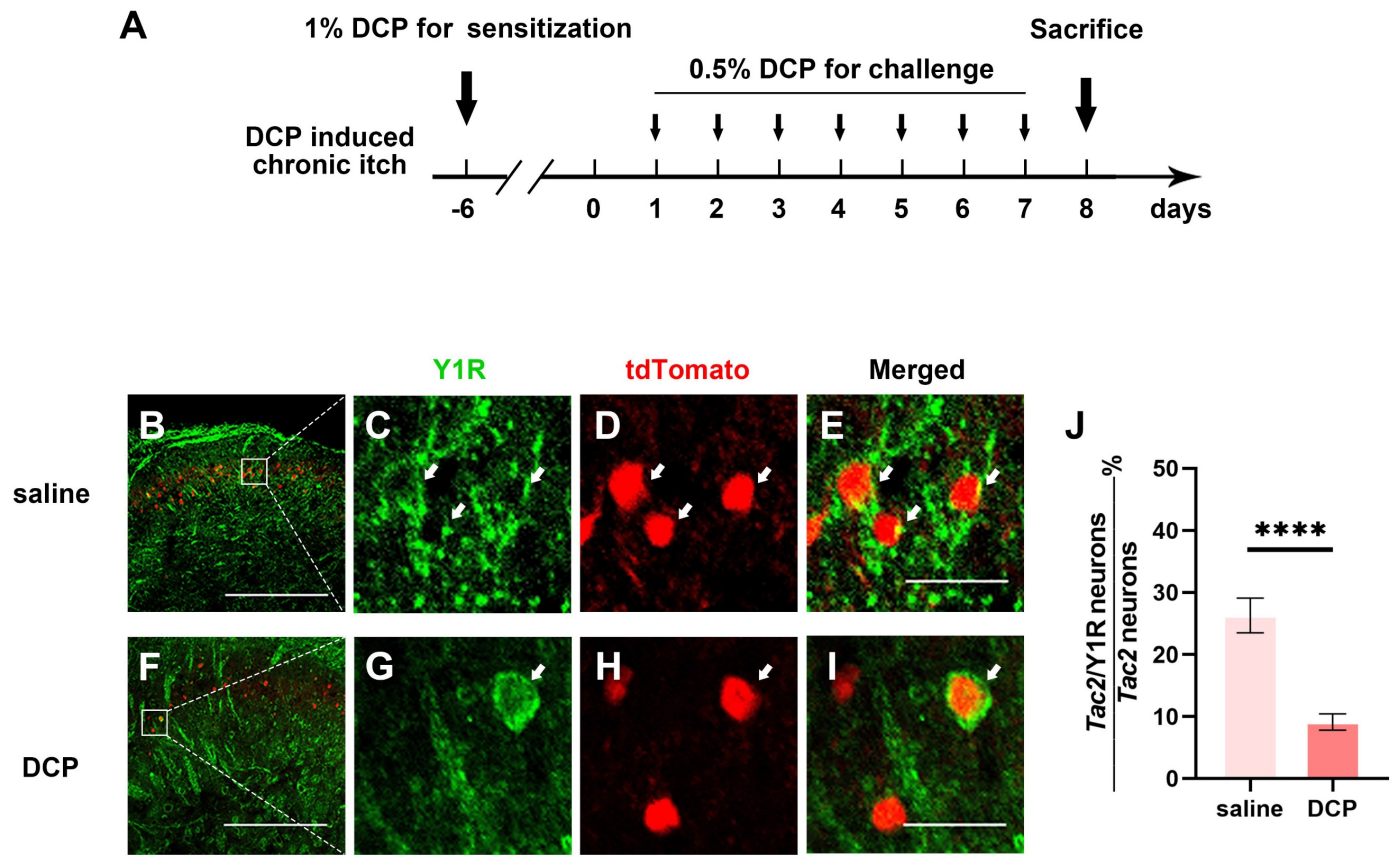
** Y1R expression on *Tac2* neurons decreases during chronic itch.** (A) A timeline of DCP model construction. (B-I) Expression of Y1R in *Tac2-*tdTomato neurons in the saline and DCP groups by immunostaining. Arrows indicate double-labeled neurons. Scale bars, 200 μm in (B) and (F), 20 μm in (C-E) and (G-I). (J) The ratio of* Tac2* and Y1R double positive neurons in *Tac2* neurons in saline (26.3 ± 2.8%) group and DCP (9.1 ± 1.3%) group. n_saline_ = 545 neurons, n_DCP_ = 492 neurons; *p* < 0.0001, Chi-square test.

Since spinal Y1R-expressing neurons have also been reported to transmit mechanical itch 23, and *Npy* neurons directly connect to mechanical itch-transmitting *Tac2* neurons, the expression of the Y1Rs on *Tac2* neurons is heavily implied. Furthermore, growing evidence suggests sensitization of mechanical itch circuits is one of the key characteristics of chronic itch 9, 10. Thus, we wondered whether Y1Rs, especially those on *Tac2* neurons, present plasticity of expression and function during chronic itch. We employed an animal model of atopic dermatitis by repeatedly painting diphenylcyclopropenone (DCP) to the nape skin, as previously described 24 (Figure 3A). First, we figured out the expression of Y1Rs on* Tac2* neurons in the saline group mice with the saline application rather than DCP. By crossing *Tac2^cre^* mice with Ai9 reporter mice, we visualized *Tac2* neurons with tdTomato. We performed immunofluorescence double staining with Y1R antibody in the cervical spinal cord of adult *Tac2^tdTomato^* mice. About 24.6% (134/545) of *Tac2* neurons overlapped with Y1R signals in saline group (Figure 3B-E). Notably, compared with the saline group, Y1R expression on *Tac2* neurons significantly decreased in the DCP group (9.6%, 47/492, *p* < 0.0001) (Figure 3F-J). Similar alterations were also noted in the lumbar spinal cord of mice with an itch model induced by topically applying DCP onto their backs (Figure S2). These suggest that *Npy* neurons may exert inhibitory effects through Y1Rs on *Tac2* neurons, and the decreased expression of Y1Rs may decline their inhibitory effects during chronic itch.

### Fewer *Tac2* neurons respond to the activation of Y1Rs during chronic itch

**Figure 4:**
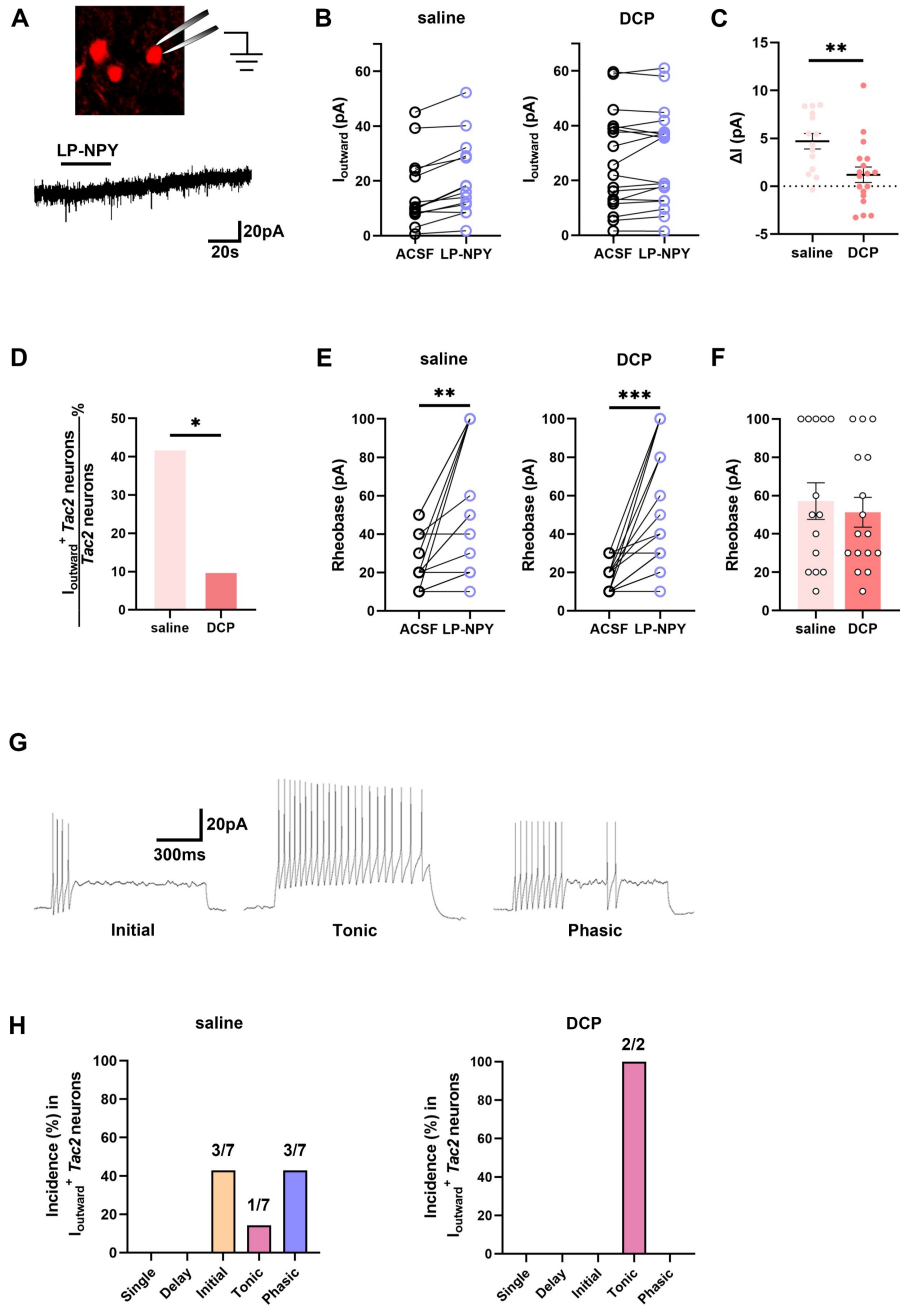
** Fewer *Tac2* neurons respond to the activation of Y1Rs during chronic itch.** (A) A schematic diagram of patch clamping a *Tac2*-tdTomato neuron (upper) and LP-NPY (1 μM, for 30 s) induced an outward current (lower) in *Tac2* neurons in voltage clamp mode at a holding potential (V_h_) of -45 mV. (B) Responses of *Tac2* neurons to LP-NPY in saline group (left) and DCP group (right). (C) Comparison of the LP-NPY induced outward current values (ΔI) in *Tac2* neurons between the saline group and the DCP group.* p* = 0.0053, two-tailed unpaired Student's *t*-test. (D) In 7 of 14 *Tac2* neurons, LP-NPY induced outward currents (ΔI ≥ 5 pA) in saline group while in 2 of 18 *Tac2* neurons in DCP group. *p* = 0.0225, Fisher's exact test. (E) The rheobases of *Tac2* neurons increased with LP-NPY administration in saline group (left) and DCP group (right). n_saline_ = 14 neurons. *p*_saline_ = 0.0019. n_DCP_ = 16 neurons. *p*_DCP_ = 0.0003, two-tailed paired Student's *t*-test. (F) Comparison of baseline rheobases in *Tac2* neurons between the saline group and the DCP group. *p* = 0.6336, two-tailed unpaired Student's *t*-test. (G) Representative traces of initial (left), tonic (middle) and phasic (right) firing pattern at 50 pA. (H) Percentages of different firing patterns of *Tac2* neurons with I_outward_ in saline group (left) and DCP group (right). n_saline_ = 7 neurons. n_DCP_ = 2 neurons.

To assess whether the function of Y1Rs on *Tac2* neurons changes during chronic itch, we performed electrophysiological recordings of *Tac2* neurons in the presence of Y1R agonist LP-NPY in saline group and DCP group respectively (Figure 4A). In saline group, LP-NPY induced outward currents in 50.0% (7/14) of *Tac2* neurons, validating the existence of functional Y1Rs on *Tac2* neurons. And these neurons were predominantly located in lamina II (7/7) rather than lamina III (0/7) of the dorsal horn of the spinal cord. Excitingly, the same dose of LP-NPY bath application induced outward currents in fewer *Tac2* neurons (11.1%, 2/18, *p* = 0.0225) in DCP group than the saline group (Figure 4B-D), even though the rheobase of* Tac2* neurons increased in both groups (Figure 4E-F). These findings indicate that the Y1R-modulated inhibition of *Tac2* neurons during chronic itch is significantly weakened. Moreover, we found that the dominating action potential firing patterns of neurons in saline group with LP-NPY-induced outward currents were initial firing (42.9%, 3/7) and phasic firing (42.9%, 3/7), followed by tonic firing (14.2%, 1/7) (Figure 4G-H). While in DCP group, the only two *Tac2* neurons with outward currents exhibited tonic firing (Figure 4H). Electrophysiological results further suggest that the inhibitory effect of* Npy* neurons upon *Tac2* neurons via Y1Rs decreased during chronic itch.

### Y1Rs-modulated synaptic transmission efficacy of *Tac2* neurons declines during chronic itch

**Figure 5:**
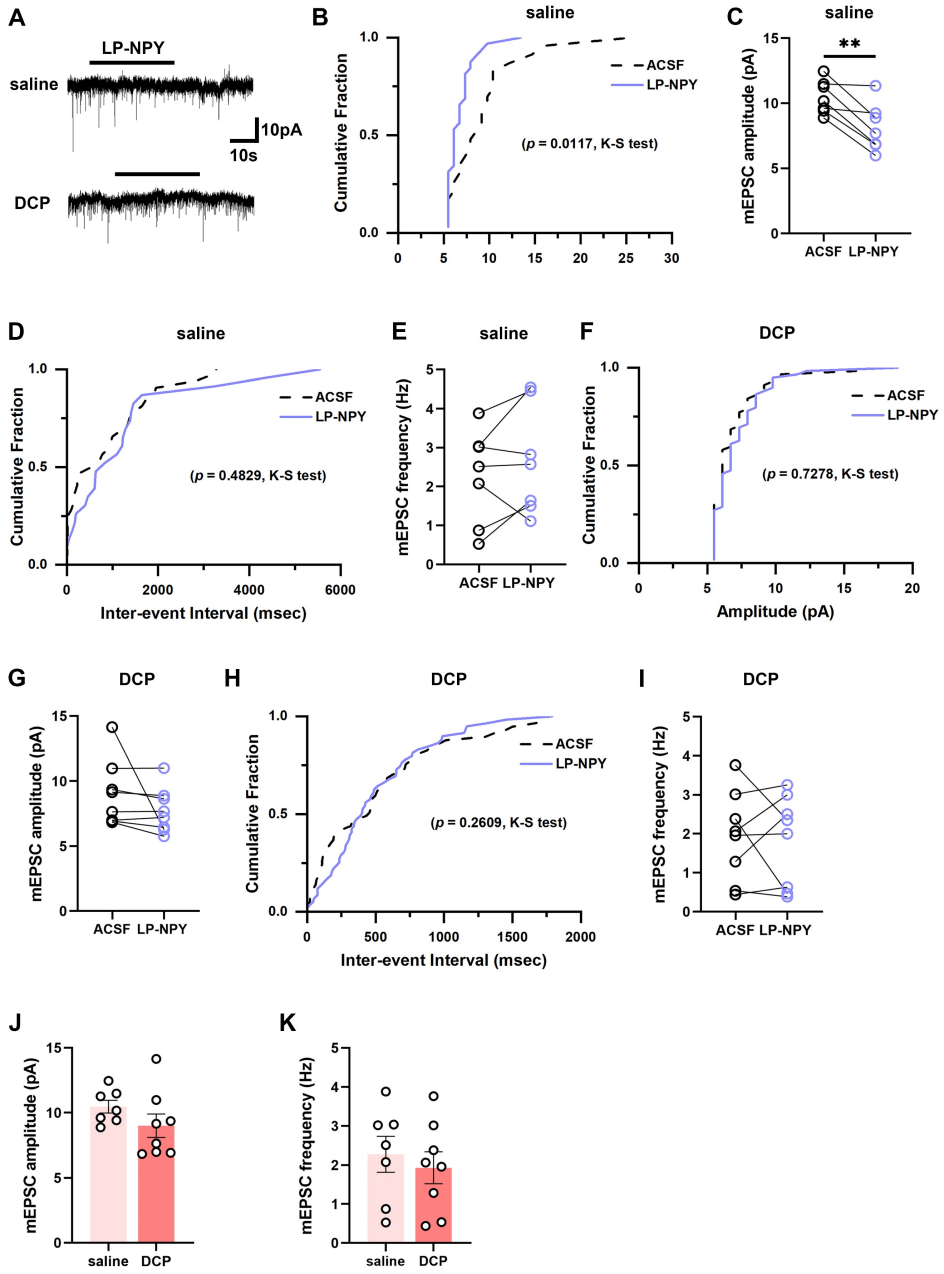
** mEPSCs on *Tac2* neurons in lamina II differ between saline and DCP mice with the application of LP-NPY.** (A) Representative traces of LP-NPY influence on mEPSC recorded in *Tac2* neurons (LP-NPY 1 μM, for 30 s, TTX 1 μM, V_h_ = -70 mV). (B) The cumulative fraction of amplitude before and after LP-NPY application. (C) Summary data showing the amplitude of mEPSCs before and after LP-NPY application. *p* = 0.0055. (D) The cumulative fraction of inter-event intervals before and after LP-NPY application. No significance. (E) Summary data showing the frequency of mEPSCs before and after LP-NPY application. No significance. *p* = 0.2609. n = 7 neurons. (B)-(E) are in saline group. (F) The cumulative fraction of amplitude before and after LP-NPY application. No significance. (G) Summary data showing the amplitude of mEPSCs before and after LP-NPY application, *p* = 0.2276. (H) The cumulative fraction of inter-event intervals before and after LP-NPY application. No significance. (I) Summary data showing the frequency of mEPSCs before and after LP-NPY application. No significance. *p* = 0.7866. n = 7 neurons. (F)-(I) are in DCP group. (J) and (K) Comparison of the amplitude (J) and frequency (K) of mEPSCs between saline group and DCP group. (C), (E), (G), (I), (J) and (K) are analyzed by two-tailed paired Student's *t*-test.

To examine the alteration of the regulatory effect of Y1Rs on the synaptic transmission efficacy of *Tac2* neurons during chronic itch, we recorded the mEPSCs and mIPSCs in spinal slices from saline and DCP groups, respectively (Figure 5A, 6A). In saline group, LP-NPY reduced the amplitude of mEPSCs of *Tac2* neurons (ACSF: 10.5 ± 0.5 pA; LP-NPY: 8.1 ± 0.7 pA; *p* = 0.0055) without affecting their frequency (ACSF: 2.3 ± 0.5 Hz; LP-NPY: 2.7 ± 0.5 Hz; *p* = 0.2609), which confirms that Y1Rs are postsynaptic and inhibitory (Figure 5B-E). Strikingly, in DCP group, LP-NPY affected neither the amplitude nor frequency (ACSF: 9.0 ± 0.9 pA, 1.9 ± 0.4 Hz; LP-NPY: 7.7 ± 0.6 pA, 1.8 ± 0.4 Hz; *p*_amplitude_ = 0.2276, *p*_frequency_ = 0.7866) (Figure 5F-I). Meanwhile, there is no difference in the amplitude (saline: 10.5 ± 0.5 pA, DCP: 9.0 ± 0.9 pA, *p* = 0.1939) or frequency (saline: 2.3 ± 0.5 Hz, DCP: 1.9 ± 0.4 Hz, *p* = 0.5825) between saline group and DCP group (Figure 5J-K).

**Figure 6:**
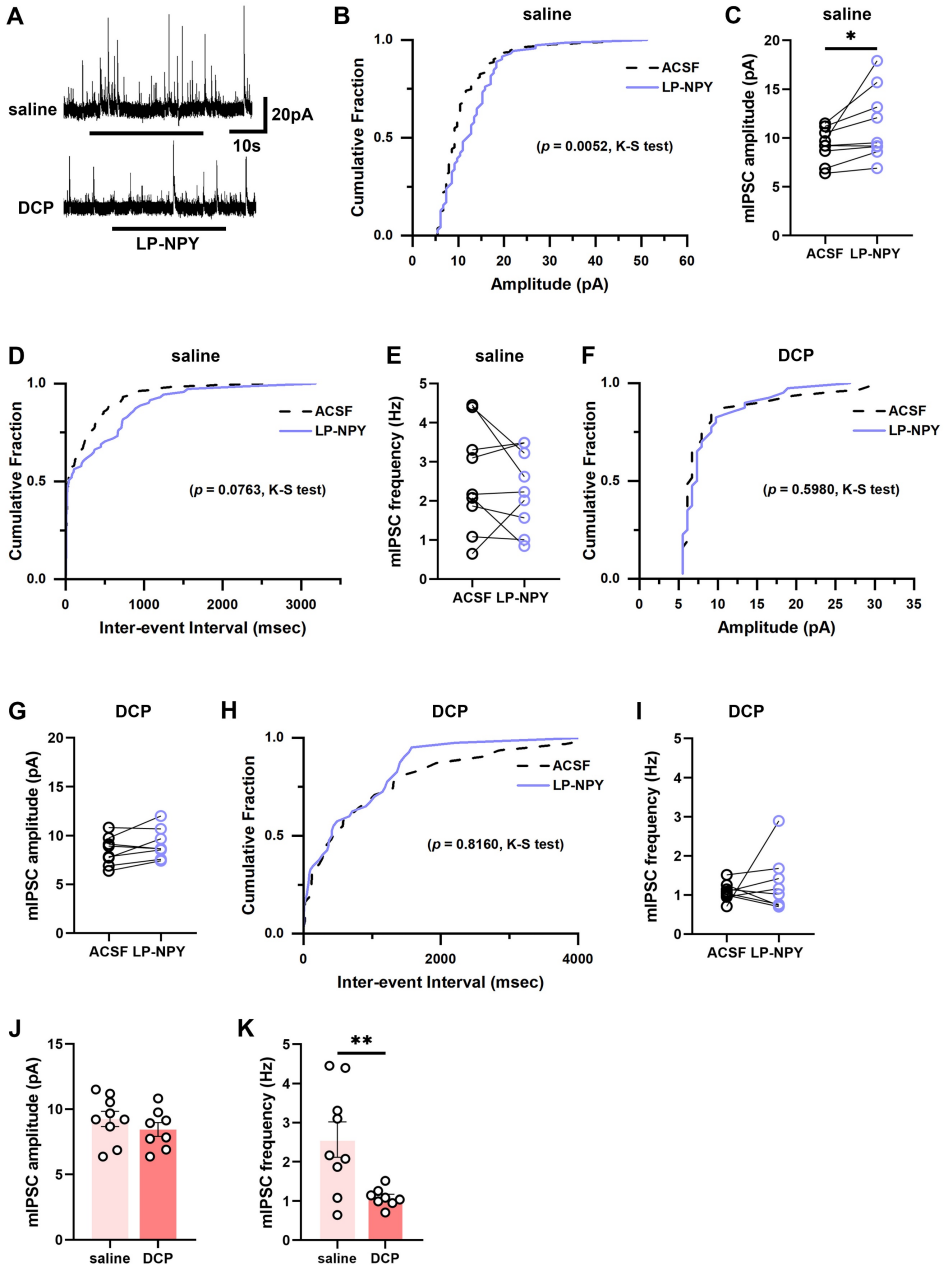
** mIPSCs on *Tac2* neurons in lamina II differ between saline and DCP mice with the application of LP-NPY.** (A) Representative traces of LP-NPY influence on mIPSCs recorded in *Tac2* neurons (LP-NPY 1 μM, for 30 s, TTX 1 μM, V_h_= 0 mV). (B) The cumulative fraction of amplitude before and after LP-NPY application. (C) Summary data showing the amplitude of mIPSCs before and after LP-NPY application. *p* = 0.0436. (D) The cumulative fraction of inter-event intervals before and after LP-NPY application. No significance. (E) Summary data showing the frequency of mIPSCs before and after LP-NPY application. No significance. *p* = 0.3959. n = 9 neurons. (B-E) are in saline group. (F) The cumulative fraction of amplitude before and after LP-NPY application. No significance. (G) Summary data showing the amplitude of mIPSCs before and after LP-NPY application. *p* = 0.0854. (H) The cumulative fraction of inter-event intervals before and after LP-NPY application. No significance. (I) Summary data showing the frequency of mIPSCs before and after LP-NPY application. No significance. *p* = 0.5086. n = 8 neurons. (F-I) are in DCP group. (J-K) Comparison of the amplitude (J) and frequency (K) of mIPSCs between saline group and DCP group. (C), (E), (G), (I), (J) and (K) are analyzed by two-tailed paired Student's *t*-test.

Similarly, we found that LP-NPY increased the amplitude of mIPSCs of *Tac2* neurons (ACSF: 9.2 ± 0.6 pA; LP-NPY: 11.3 ± 1.2 pA; *p* = 0.0436) without affecting the frequency (ACSF: 2.6 ± 0.4 Hz; LP-NPY: 2.3 ± 0.3 Hz, *p* = 0.3959) (Figure 6A-E). While in the chronic itch, LP-NPY did not affect the amplitude or frequency (ACSF: 8.4 ± 0.5 pA, 1.1 ± 0.1 Hz; LP-NPY: 9.1 ± 0.6 pA, 1.3 ± 0.3 Hz; *p*_amplitude_ = 0.0854, *p*_frequency_ = 0.5086) (Figure 6A, 6F-I). Besides, there is no difference in the amplitude between saline group and DCP group (saline: 9.2 ± 0.6 pA, DCP: 8.4 ± 0.5 pA, *p* = 0.3244) (Figure 6J). Remarkably, the frequency of mIPSC was significantly lower in DCP group than saline group (saline: 2.6 ± 0.4 Hz, DCP: 1.1 ± 0.1 Hz, *p* = 0.0080) (Figure 6K). These results demonstrate that the regulatory effect of Y1Rs on the synaptic transmission efficacy of *Tac2* neurons declines during chronic itch.

## Discussion

A previous study has shown that inhibition of *Npy* neurons induces mechanical hyperknesis in mice, which suggests that pathways transmitting mechanical itch are in a state of hyperexcitability 1. Our study found that c-Fos expression in mechanical itch-transmitting *Tac2* neurons increased upon *in vivo* inhibition of *Npy* neurons (Figure 1H-I). Additional retrograde monosynaptic tracing results also confirmed that *Npy* neurons were upstream of *Tac2* neurons and made direct synaptic connections with them (Figure 2C). Thus, *in vivo* inhibition of *Npy* neurons leads to disinhibition of *Tac2* neurons, which explains the hyperknesis in mice. In the acetone-ether-water (AEW) model, the expression of c-Fos was induced to increase in *Tac2* neurons even in the absence of von Frey stimulation 10. However, it remains to be investigated whether this is due to the declining inhibitory effect of* Npy* neurons on *Tac2* neurons.

NPY exerts its cellular regulatory effects through six subtypes of receptors 25. Among them, Y1Rs and Y2Rs are predominantly expressed in the spinal cord 26, 27. Y1Rs are mainly postsynaptic and Y2Rs are presynaptic 27, 28. Y1Rs are coupled to Gi protein, and when NPY binds to Y1Rs to activate Gi protein, the AC/cAMP/PKA pathway is inhibited, followed by the inactivation of cation channels, normally activated by excitatory neurotransmitters such as glutamate, and finally neuronal excitability is suppressed 29-31. Thus, Y1Rs on the pathway that transmits mechanical itch play a crucial role in the negative regulatory effect of *Npy* neurons. Alterations in the expression and function of Y1Rs can directly affect mechanical itch transmission. We found that Y1Rs are positively expressed on* Tac2* neurons (Figure 3B-E), which provides an anatomical basis for the *Npy* neurons regulating mechanical itch transmission via the NPY-Y1R system. In addition, we found that Y1Rs expression on *Tac2* neurons significantly decreased in the chronic itch state (Figure 3F-J). This reduction results in the inability of NPY to modulate *Tac2* neurons, as confirmed by subsequent electrophysiological studies.

In addition, we classified the *Tac2* neurons that respond to LP-NPY by their location and action potential pattern. First, we found that these neurons were predominantly located in lamina II (7/7) rather than lamina III (0/7) of the dorsal horn of the spinal cord, which suggests that *Tac2* neurons in the lamina II expressing Y1Rs may be the same group of neurons as *Tac2* neurons in the lamina II with increased activity identified in the AEW chronic itch model 10. Second, this group of *Tac2* neurons was predominantly characterized by initial (3/7) and phasic (3/7) action potential firing patterns (Figure 4F). Since previous studies have shown that low-threshold cells that respond only to innocuous stimuli invariably exhibit a phasic firing pattern 32, and *Tac2* neurons receive LTMR Aβ fiber afferents 10, *Tac2* neurons distributed in the lamina II that expresses the Y1Rs and have phasic firing pattern are more likely to be responsible for chronic itch. In the DCP model, the proportion of *Tac2* neurons that responded to LP-NPY significantly decreased (Figure 4B-C), which is consistent with the decreased Y1Rs expression. By the way, the only two neurons in the DCP group that responded to LP-NPY exhibited tonic firing (Figure 4F). Nonetheless, it is not yet reasonable to conclude that *Tac2* neurons with different firing patterns in the DCP model do not respond to LP-NPY due to the limited number of neurons recorded.

NPY acts as a neuromodulator and indirectly regulates neurotransmitter signaling through G protein-coupled receptors 33. Presynaptic receptors, such as Y2Rs, regulate neurotransmitter release, while postsynaptic receptors, such as Y1Rs, modulate neurotransmitter-gated ion channels, such as NMDAR, AMPAR, GABAR and glycine receptors, further affecting the efficacy of synaptic transmission of glutamate, GABA or glycine neurotransmitters 34, 35. Thus, during chronic itch, is the efficacy of NPY-Y1R-mediated regulation of glutamatergic, GABAergic, and glycinergic synaptic transmission on *Tac2* neurons altered due to decreased Y1Rs expression? Through electrophysiological studies, we found that under normal conditions, activation of the Y1Rs decreased the mEPSCs amplitude and left the frequency unchanged, whereas the mIPSCs amplitude increased and the mIPSCs frequency remained unchanged (Figure 5 and Figure 6). This may be because Y1Rs specifically recruit Gi protein upon binding LP-NPY, which inhibits adenylyl cyclase (AC), and subsequently reduces the intracellular concentration of cAMP 33, 36. Moreover, a reduction of cAMP will decrease NMDAR-mediated currents 37-39 and enhance GABAR-mediated currents 40, finally leading to a decrease in mEPSCs amplitude and an increase in mIPSCs amplitude 30, 34. However, the presynaptic neurotransmitter release was not altered, thus the frequency of mEPSCs and mIPSCs remained unchanged 41. Previous studies reported that NPY had no effect on the amplitude and attenuated the frequency of mEPSCs and mIPSCs or not in spinal cord dorsal horn substantia gelatinosa (SG) neurons 28, 31, 34, 41, which appears to be different from our results. However, we believe that there are at least two reasons for this discrepancy: on the one hand, we used a specific Y1R agonist to selectively activate the Y1Rs, whereas they perfused NPY, which activates not only the Y1Rs but also the Y2Rs. Thus, their recorded mEPSCs and mIPSCs may be the result of a mixed effect of Y1Rs and the Y2Rs; on the other hand, we selectively recorded *Tac2* neurons by cross-breeding *Tac2^cre^* mice with Ai9 reporter mice and visualizing *Tac2* neurons with tdTomato. However, they randomly recorded neurons in SG. As there are a wide variety of cell types in the SG, of which a large number are inhibitory 42, the statistical differences are likely masked by the fact that these distinct neuron types respond differently to NPY. We selectively studied *Tac2* neurons and more precisely observed their response to the activation of Y1Rs. In addition, we found that there was no significant difference in the amplitude or frequency of mEPSCs and the amplitude of mIPSCs in the saline and DCP groups, but the frequency of mIPSCs in the DCP group was significantly lower than that in the saline group, indicating that NPY release from the presynaptic membrane or that direct synaptic connections between *Npy* neurons and* Tac2* neurons reduce during chronic itch.

**Figure 7:**
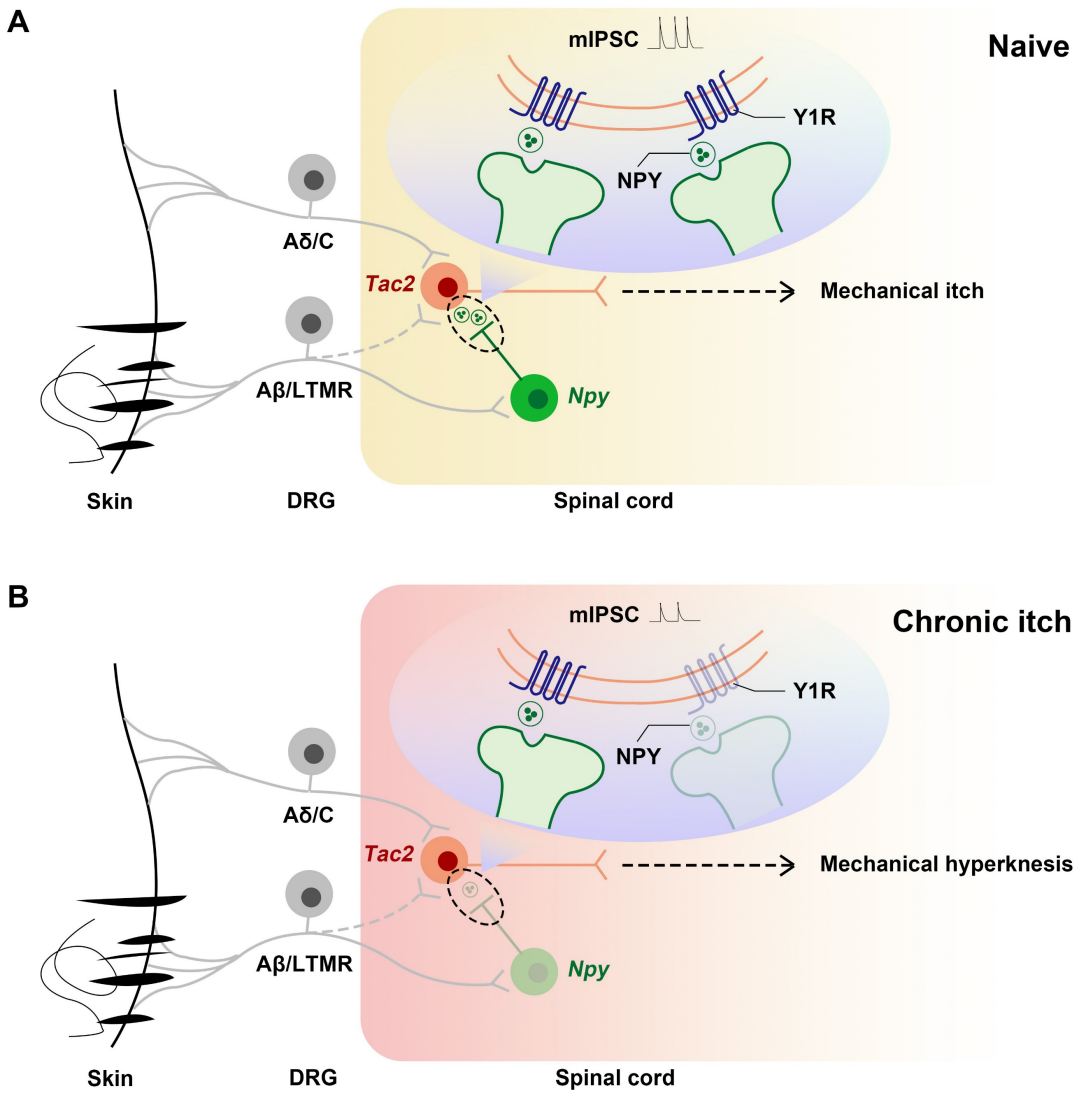
** Schematics showing the alteration of the NPY-Y1R system between *Tac2* and *Npy* neurons.** (A) Under naive condition, *Tac2* neurons receive innocuous light touch information via Aβ/LTMR fibers to transmit mechanical itch, gated by feedforward inhibition of *Npy* neurons. (B) Due to decreased Y1Rs and reduced presynaptic NPY release or direct synaptic connections between *Npy* neurons and *Tac2* neurons, a loss of feedforward inhibition mediated by *Npy* neurons (reduced frequency and amplitude of mIPSC) gives rise to mechanical hyperknesis during chronic itch. The dotted lines represent indirect inputs to *Tac2* neurons. ​The dashed lines represent indirect projections.

In summary, we demonstrate that, during chronic itch, decreased expression of Y1Rs on *Tac2* neurons, a key neuron in the transmission of mechanical itch, leads to decreased inhibition of *Npy* neurons to* Tac2* neurons, increased activity of *Tac2* neurons, decreased efficacy of inhibitory synaptic transmission, and increased efficacy of excitatory synaptic transmission. These alterations predispose the afferents to evoke mechanical itch signaling, giving rise to mechanical hyperknesis (Figure 7).

So far, growing evidence supports the inhibitory role of NPY and Y1Rs in itch. Lack of spinal NPY is involved in mechanical hyperknesis in aged mice, and specific ablation or silence of spinal *Npy* neurons or conditional knockout of spinal Y1Rs in mice develop mechanical hyperknesis 1, 22, 23. In this study, we found a significant reduction of Y1Rs on *Tac2* neurons in the dorsal horn of the spinal cord during chronic itch. Considering the finding that the lack of spinal NPY is involved in mechanical hyperknesis in aged mice, we speculate that the simultaneous reduction of NPY and Y1Rs expression exists during chronic itch (the reduced mIPSCs frequency in the DCP group compared with that in the saline group also indicates that presynaptic NPY release or that direct synaptic connections between *Npy* neurons and *Tac2* neurons reduce during chronic itch). Quantifying the local NPY in the terminals of spinal *Npy* neurons, as well as around mechanical itch-transmitting neurons during chronic itch, would give us additional direct evidence.

Compared with itch, the role of NPY and Y1Rs in pain is more ambiguous. First, it remains unclear whether NPY is analgesic or nociceptive. Previous studies have reported that intrathecal injection of NPY had an antinociceptive effect 15, 18, 43. Although ablation and silence of spinal *Npy* neurons did not develop hyperalgesia 1, a recent study reported that chemogenetic activation of *Npy* neurons not only increased acute nociceptive thresholds but also rescued hyperalgesia in inflammatory and neuropathic pain 21. But it has also been claimed that NPY intrathecal injection causes mechanical hyperalgesia 44, and subcutaneous injection of NPY exacerbates mechanical and thermal hyperalgesia 45. Second, the relationship between Y1Rs and pain remains elusive. Some findings favor their analgesic role. In chronic inflammatory pain models, Y1Rs expression increased 46. Y1R agonist alleviates thermal hyperalgesia 45 and Y1R antagonist reverses the antinociceptive effect of intrathecally injected NPY 18. Y1Rs knockout mice exhibit marked mechanical hypersensitivity 14. However, Y1Rs have also been reported to be involved in nerve injury-induced mechanical hyperalgesia 44. Although it is not clear whether NPY and Y1Rs play analgesic or nociceptive roles, respectively, it is certain that *Npy* neurons respond to both nociceptive and innocuous mechanical stimuli 1, 16, and that NPY and Y1Rs in the dorsal horn of the spinal cord are significantly upregulated during nerve injury and inflammation 17, 46, 47. In contrast, it is likely that both NPY and Y1Rs expressionare reduced simultaneously during chronic itch. Inverse changes in NPY and Y1Rs during chronic itch and chronic pain may suggest, to some extent, that there is a difference in gate regulation between chronic pain and chronic itch. The initiating cause for the disinhibition of the spinal excitatory interneurons that causes chronic pain may not be NPY-Y1R system but other spinal inhibitory interneurons.

Our study not only further elucidates the mechanism of action of NPY-Y1R system in modulating mechanical itch, but also provides a therapeutic target for mechanical hyperknesis during chronic itch. Y1R agonists would be a promising alternative treatment for chronic itch. However, it remains a challenge to develop Y1R agonists that selectively act on *Tac2* neurons without affecting nociception and tactile sensations. Meanwhile, it needs further investigation whether synapses or NPY in the synapses between* Npy* neurons and* Tac2* neurons decrease during chronic itch.

## Supplementary Material

Supplementary figures.
