# Supplementary material for: The plasticity of neuropeptide Y-Y1 receptor system on Tac2 neurons contributes to mechanical hyperknesis during chronic itch
Source: Theranostics. 2024 Jan 1;14(1):363–78. doi: 10.7150/thno.89433 (PMC10750199; doi:10.7150/thno.89433)
Supplement: Supplementary file 1 — Supplementary figures. [file thnov14p0363s1.pdf]

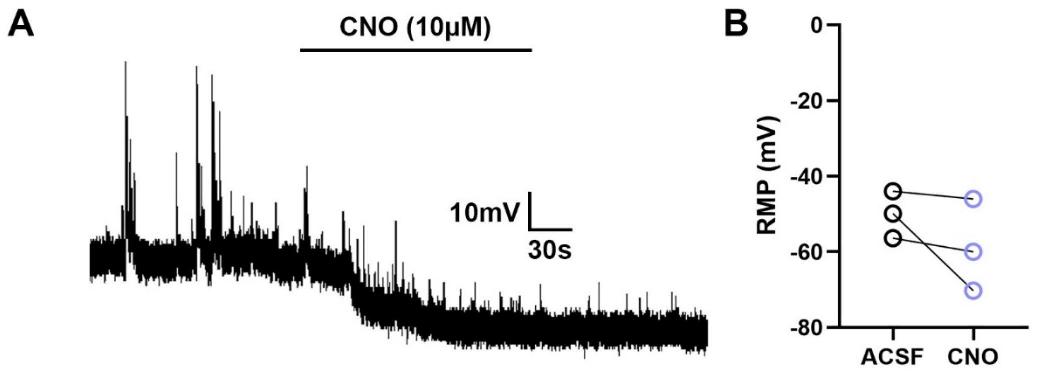

Figure S1. Functional efficacy of AAV2/9-hSyn-fDIO-hM4Di-mCherry (A) Bath application of CNO (10 μM) induced hyperpolarization of a hM4Di-mCherry neuron in the spinal cord. (B) Summary graph showing the resting membrane potential (RMP) before and after CNO application (n = 3 from 2 mice).

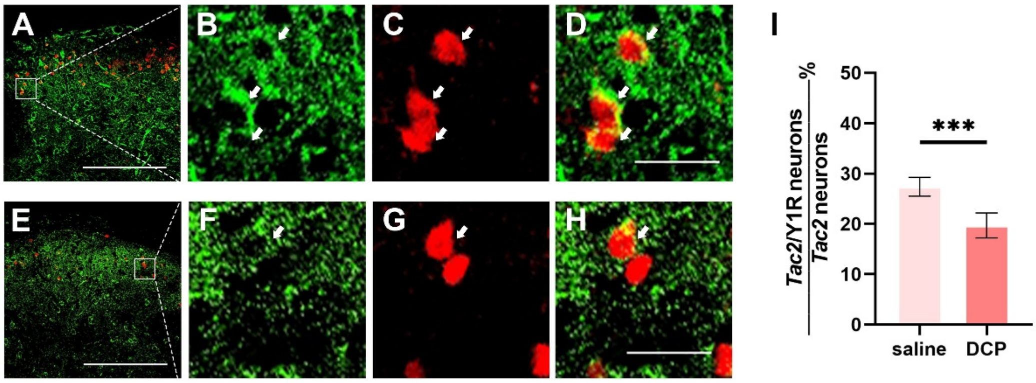

Figure S2. Y1R expression on *Tac2* neurons in lumbar spinal cord decreases during chronic itch. (A-H) Expression of Y1R in *Tac2*-tdTomato neurons in the saline and DCP groups by immunostaining. Arrows indicate double-labeled neurons. Scale bars, 200 µm in (A) and (E), 20 µm in (B-D) and (F-H). (I) The ratio of *Tac2* and Y1R double positive neurons in *Tac2* neurons in saline group and DCP group. $n_{saline} = 539$ neurons, $n_{DCP} = 494$ neurons; $p = 0.0009$, Chi-square test.
